# Using Principles of an Adaptation Framework to Adapt a Transdiagnostic Psychotherapy for People With HIV to Improve Mental Health and HIV Treatment Engagement: Focus Groups and Formative Research Study

**DOI:** 10.2196/45106

**Published:** 2023-05-30

**Authors:** Doyanne Darnell, Minu Ranna-Stewart, Christina Psaros, Teresa R Filipowicz, LaKendra Grimes, Savannah Henderson, Mariel Parman, Kathy Gaddis, Bradley Neil Gaynes, Michael J Mugavero, Shannon Dorsey, Brian W Pence

**Affiliations:** 1 University of Washington Seattle, WA United States; 2 Massachusetts General Hospital Boston, MA United States; 3 University of North Carolina at Chapel Hill Chapel Hill, NC United States; 4 University of Alabama at Birmingham Birmingham, AL United States

**Keywords:** adaptation, transdiagnostic psychotherapy, people with HIV, trauma, comorbidity

## Abstract

**Background:**

HIV treatment engagement is critical for people with HIV; however, behavioral health comorbidities and HIV-related stigma are key barriers to engagement. Treatments that address these barriers and can be readily implemented in HIV care settings are needed.

**Objective:**

We presented the process for adapting transdiagnostic cognitive behavioral psychotherapy, the Common Elements Treatment Approach (CETA), for people with HIV receiving HIV treatment at a Southern US HIV clinic. Behavioral health targets included posttraumatic stress, depression, anxiety, substance use, and safety concerns (eg, suicidality). The adaptation also included ways to address HIV-related stigma and a component based on Life-Steps, a brief cognitive behavioral intervention to support patient HIV treatment engagement.

**Methods:**

We applied principles of the Assessment, Decision, Administration, Production, Topical Experts, Integration, Training, Testing model, a framework for adapting evidence-based HIV interventions, and described our adaptation process, which included adapting the CETA manual based on expert input; conducting 3 focus groups, one with clinic social workers (n=3) and 2 with male (n=3) and female (n=4) patients to obtain stakeholder input for the adapted therapy; revising the manual according to this input; and training 2 counselors on the adapted protocol, including a workshop held over the internet followed by implementing the therapy with 3 clinic patients and receiving case-based consultation for them. For the focus groups, all clinic social workers were invited to participate, and patients were referred by clinic social workers if they were adults receiving services at the clinic and willing to provide written informed consent. Social worker focus group questions elicited reactions to the adapted therapy manual and content. Patient focus group questions elicited experiences with behavioral health conditions and HIV-related stigma and their impacts on HIV treatment engagement. Transcripts were reviewed by 3 team members to catalog participant commentary according to themes relevant to adapting CETA for people with HIV. Coauthors independently identified themes and met to discuss and reach a consensus on them.

**Results:**

We successfully used principles of the Assessment, Decision, Administration, Production, Topical Experts, Integration, Training, Testing framework to adapt CETA for people with HIV. The focus group with social workers indicated that the adapted therapy made conceptual sense and addressed common behavioral health concerns and practical and cognitive behavioral barriers to HIV treatment engagement. Key considerations for CETA for people with HIV obtained from social worker and patient focus groups were related to stigma, socioeconomic stress, and instability experienced by the clinic population and some patients’ substance use, which can thwart the stability needed to engage in care.

**Conclusions:**

The resulting brief, manualized therapy is designed to help patients build skills that promote HIV treatment engagement and reduce symptoms of common behavioral health conditions that are known to thwart HIV treatment engagement.

## Introduction

### Background

**Figure 1:**
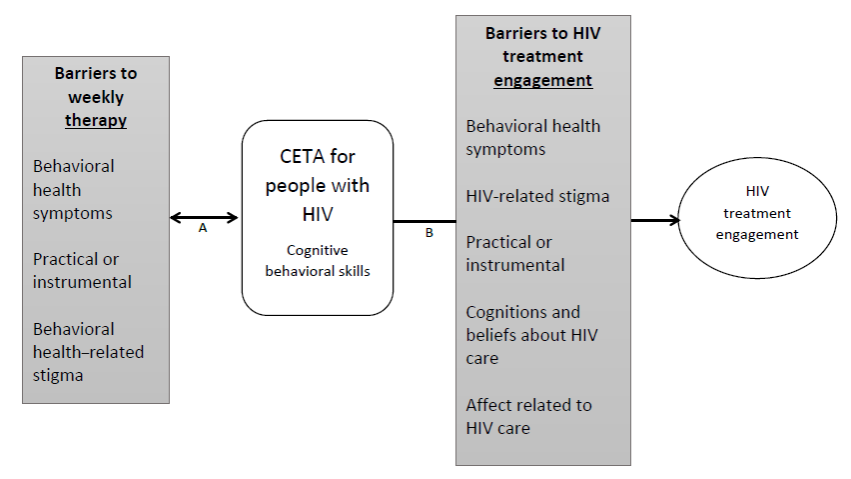
Conceptual model of the Common Elements Treatment Approach (CETA) for people with HIV. (A) CETA for people with HIV components address barriers to attending weekly sessions once patients initiate the therapy. (B) CETA for people with HIV components address barriers to HIV treatment engagement through attendance to weekly sessions.

HIV treatment engagement, which includes taking antiretrovirals as prescribed and attending routine medical appointments with an HIV treatment provider, is critical for people with HIV. Intermittent HIV treatment engagement can severely limit the effectiveness of antiretroviral therapy and reduces protection against transmission of the virus to other people. Psychiatric comorbidity is common among people with HIV, associated with poor HIV treatment engagement and worse HIV treatment outcomes. Integrated treatment for mental health and substance use disorders within HIV care is recommended to best serve the needs of this population and improve HIV treatment engagement and outcomes. In this paper, we present our process for adapting a multiproblem transdiagnostic cognitive behavioral therapy, the Common Elements Treatment Approach (CETA), a manualized yet flexible therapy to address symptoms of depression, anxiety, posttraumatic stress, and substance use problems, to improve mental health and HIV treatment engagement among patients receiving HIV treatment in a US HIV clinic (Figure 1). First, we provide background on the need for such a model and the potential value of CETA—or other transdiagnostic or multiproblem interventions—in meeting this need. We then present our adaptation steps, informed by the Assessment, Decision, Administration, Production, Topical Experts, Integration, Training, Testing (ADAPT-ITT) model, and case presentations to illustrate the use, challenges, and potential benefits of the adaptation of CETA for people with HIV.

### The Need to Address Behavioral Health Conditions to Support Patients in HIV Treatment Engagement

Depression is the most common psychiatric comorbidity among people with HIV, affecting an estimated 20% to 40% of the population [1], and is associated with antiretroviral treatment nonadherence [2], missed HIV appointments, virologic failure, and higher mortality rates [3]. Depression among people with HIV often co-occurs with anxiety, posttraumatic stress, or substance use disorders [4]. Of 304 patients with HIV enrolled in a depression treatment trial in the southern United States, all of whom met the criteria for a current major depressive episode, 62% also had one or more anxiety disorder diagnoses, and 28% had a current substance use disorder [4].

There is evidence supporting the efficacy of standard pharmacological and psychotherapeutic interventions for depression, anxiety, posttraumatic stress, and substance use disorders among people with HIV [5,6]; however, there are challenges to implementing evidence-based psychotherapy routinely in this context. The typical approach is to train therapists in manualized protocols that target symptom clusters underlying specific disorders, such as separate protocols for depression, anxiety, posttraumatic stress, or substance use problems. Importantly, these protocols may not be tailored to the priority population, which often has comorbid features. This narrow focus on one disorder at a time at the expense of co-occurring disorders may increase the risk of treatment failure [7,8] and places considerable demand on already limited behavioral health care provider and clinic resources.

### CETA Therapy

The CETA was originally developed as an evidence-based, flexible, modular transdiagnostic psychotherapy treatment approach designed to address the needs of individuals with comorbid mental health conditions and be delivered by lay counselors in low- and middle-income countries (LMICs) [9]. The interest in developing a transdiagnostic model stemmed from several limitations of singular diagnostic-focused treatments, including few options or guidance for flexibility when a wider range of client mental health symptoms are present and lack of resources to support training and implementation of multiple evidence-based treatments in LMICs for different diagnostic targets. Owing to limited professional mental health care providers in LMICs, lay counselors, who had little to no previous mental health training or experience delivering therapy, were trained to deliver CETA under supervision [10], which involved using simplified treatment language and providing lay counselors with in-session supports (eg, specific steps to be taken in the session outlined and described for counselors).

The initial development of CETA for LMICs focused on treating common mental health comorbidities among adult trauma survivors in LMICs, such as posttraumatic stress, anxiety, and depression, with demonstrated effectiveness in treating these symptoms. In a randomized trial in southern Iraq with survivors of systematic violence (including torture), CETA (n=99) demonstrated large effect sizes for posttraumatic stress (Cohen *d*=2.40), anxiety (Cohen *d*=1.60), and depression (Cohen *d*=1.82) symptoms relative to a waitlist control (n=50) [11]. A total of 2 adverse events were reported in this study, with one patient dying by suicide after the initial CETA session and another patient being hospitalized for depression and subsequently dropping out of CETA. In a randomized trial with Burmese refugees in Thailand (N=347), CETA (n=182) demonstrated large effect sizes for posttraumatic stress (Cohen *d*=1.19), depression (Cohen *d*=1.16), and anxiety (Cohen *d*=0.79) relative to a waitlist control (n=165) [10]. There were no adverse events observed or reported in this study. Later, CETA incorporated a component targeting alcohol use, which demonstrated efficacy in a pilot randomized trial with adults living with HIV in Zambia [12]. Specifically, participants in the condition with CETA plus a brief alcohol intervention component (n=82) reported reduced alcohol use over 6 months compared with participants in a brief alcohol intervention–only condition (n=78), resulting in a medium effect size (Cohen *d*=0.48). No adverse events were observed or reported in this study.

CETA incorporates principles of cognitive behavioral therapy and measurement-based care—the systematic evaluation of patient symptoms before or during an encounter to inform behavioral health treatment—to target depression, anxiety, posttraumatic stress, and unhealthy substance use. The clinician first assesses the patient’s symptoms in these areas using standardized measures and then uses these assessments, patient goals, and clinical judgment to determine which therapeutic components to deliver (eg, components to reduce unhelpful thoughts or avoidance behavior; refer to Textbox 1 for the CETA components). Additional components to address safety concerns, including suicidality, homicidality, and domestic violence, are also available to be used at any time during therapy.

CETA is typically delivered in a one-on-one format for approximately an hour per session (typically one component per session) and typically lasts 6 to 12 weeks depending on the complexity of the patient’s clinical presentation. CETA sessions follow the structure of cognitive behavioral therapy and include agenda setting, skill instruction and practice, and assigning and reviewing homework based on applying skills learned in the patient’s daily life. The therapy plan is updated as needed, and the “dose” or number of sessions for a particular component are decided upon through repeated administration of standardized measures of the clinical targets, client interview and preferences, and clinical judgment based on case conceptualization. Patients may initially experience an increase in symptoms that decrease over time during therapy as a consequence of reducing avoidance of stressful situations and painful memories.

CETA is a promising patient-centered psychotherapeutic approach to meet the mental health and substance use needs of patients receiving care in US-based HIV clinics. Although it is a manualized and replicable therapy, it is also flexible enough to respond to the needs of a wide range of patients presenting with a range of disorders [13]. Furthermore, the structure of the therapy, which relies on the selection of various treatment components (as described in the following sections), lends itself to being adapted to also target HIV treatment engagement and apply skills to the unique needs of people with HIV (eg, HIV-related stigma). Finally, given that CETA was developed specifically to be deployed in low-resource settings by therapists representing a range of clinical experience and training, we anticipate that existing clinic HIV care providers will be able to uptake and deliver the treatment with fidelity. Although CETA has not previously been deployed in US-based HIV clinics, it has been implemented with promising initial data in US public behavioral health clinics [14] and successfully deployed outside the United States in HIV clinics in Lusaka, Zambia, by clinic peer educators [12].

**Common Elements Treatment Approach (CETA) for people with HIV targets and components listed in order of delivery (initial sessions, middle sessions, and the final session). The Life-Steps component was added to CETA for CETA for people with HIV.**

CETA for people with HIV

- Psychotherapy for clinical targets: HIV treatment engagement, HIV-related stigma, and common behavioral health conditions (depression, anxiety, posttraumatic stress, and substance use).

Initial sessions

- The first 2-3 sessions include orienting the patient to CETA for people with HIV and completing the Life-Steps component.
  - Encouraging participation
    - Complete standardized symptom assessments. Provide overview of CETA for people with HIV and the cognitive behavioral therapeutic approach. Troubleshoot practical barriers to engaging in CETA for people with HIV. Encouraging Participation is delivered as a component that is the focus of an entire session but may also be returned to at any time in therapy as needed.
  - Introduction
    - Review the patient’s symptoms and goals for therapy and provide psychoeducation about how CETA for people with HIV will address the patient’s unique symptoms and goals.
  - Life-steps
    - Provide psychoeducation about HIV treatment engagement. Identify patient’s goals regarding HIV treatment engagement and strategies to overcome practical barriers to treatment engagement. Additional and more complex barriers are addressed using skills learned in other CETA for people with HIV components.

Middle sessions

- The following components are selected and ordered based on the patient’s CETA for people with HIV goals and clinical targets:
  - Thinking in a different way part I
    - Skills for modifying negative unhelpful automatic thoughts to more helpful thoughts
    - Potential targets: all
  - Thinking in a different way part II
    - Skills for modifying negative unhelpful beliefs to something more helpful
    - Potential targets: all
  - Relaxation
    - Skills to reduce physiological arousal and tension
    - Potential targets: anxiety, posttraumatic stress, substance use reduction, and HIV treatment engagement
  - Getting active
    - Skills for scheduling activities to increase engagement in enjoyable or meaningful activities
    - Skills for effective goal setting
    - Potential targets: depression and HIV treatment engagement goals
  - Live exposure
    - Skills for reducing avoidance of anxiety-provoking or feared but objectively safe or nonthreatening situations
    - Potential targets: anxiety, posttraumatic stress, perceived HIV-related stigma, and HIV treatment engagement
  - Talking about difficult memories
    - Skills for reducing avoidance of traumatic memories
    - Potential targets: posttraumatic stress
  - Substance use reduction
    - Skills for reducing substance use; includes skills for increasing motivation for health behaviors and assertive communication
    - Potential targets: substance use reduction, perceived or experienced HIV-related stigma, and HIV treatment engagement
  - Solving problems
    - Skills for solving problems or reducing stressors
    - Potential targets: all
  - Safety
    - Address safety concerns related to suicide, homicide, and interpersonal violence, including safety planning

Final session: finishing steps

- In the final session, the clinician and patient review the skills the patient has learned in CETA for people with HIV, and the patient is encouraged to continue using the skills as needed.

## Methods

### Ethics Approval

The study was approved by the University of Alabama at Birmingham Institutional Review Board (IRB-300004217).

### Informed Consent

All participants completed informed consent procedures. Steps were taken to protect participants’ privacy and confidentiality, including secure storage of data, use of Health Insurance Portability and Accountability Act–compliant videoconferencing software, deidentification of data for analysis and data retention, and masking of patient information presented in our case example. Persons from vulnerable populations were not recruited.

### Participation

Participants in the patient focus groups were compensated with US $50; participants in the provider focus group received no compensation. Participants receiving CETA for people with HIV were not compensated for attending therapy sessions.

### Clinical Setting

The study clinic, located in the southern United States, has provided comprehensive HIV medical and social services for >25 years and sees >4000 patients annually. Nearly all patients at the clinic complete patient-reported outcomes every 4 to 6 months using validated scales, including depression and panic symptom assessments. CETA for people with HIV was integrated into existing mental health and social services such that study counselors were social workers otherwise providing services as usual to patients at the clinic and patients continued to receive HIV treatment as usual at the clinic.

### Steps for Adapting CETA for People With HIV to Improve HIV Treatment Engagement

#### Overview

The adaptation of CETA for people with HIV was informed by the ADAPT-ITT model developed by Wingood and DiClemente [15] as a framework to ensure successful adaptation of evidence-based interventions for HIV and ensure that adaptations are culturally sound and acceptable to their target populations. Specifically, our team used principles that underlie the model, including the following: (1) adaptation should be collaborative and participatory, incorporating opinions and perspectives of all stakeholders to identify what is needed and how well it is working; (2) adaptation should be based on empirical information and rigorous study designs; and (3) adaptation should be an iterative process. We also used suggested methodologies such as collecting and analyzing qualitative data to obtain stakeholder input on the proposed intervention and will complete pilot-testing of the intervention. We largely followed the sequential phase approach of the model but at times used alternative methodology to what is described in the ADAPT-ITT model article to complete the phase [15]. For instance, although we explored the questions that are consistent with the administration phase (*what in the original intervention needs to be adapted and how should it be adapted?*), we did not use “theater testing” (ie, demonstration of the intervention) but focus groups to capture stakeholder input.

The goal was to modify CETA to improve HIV treatment engagement among people with HIV with behavioral health conditions who were also demonstrating or at increased risk of poor HIV treatment engagement. A team of experts in the treatment of HIV, CETA, and intervention development adapted CETA for people with HIV with input from stakeholder groups. These groups included people with HIV with experience with psychotherapy, providers at an HIV clinic who delivered psychotherapy, and clinic supervisory staff. Our adaptation process unfolded over 4 steps.

#### Adaptation Step 1: Develop the Initial CETA for People With HIV Manual

##### Overview

Once CETA was identified as a promising therapeutic approach for adaptation to target HIV treatment engagement, the study team revisited the literature on behavioral health and other factors that interfere with HIV treatment engagement to ensure that important clinical targets and components were included. From this process, we added HIV-related stigma (internalized and externalized) as a clinical target based on the high rates of stigma experienced among people with HIV and its known negative impact on behavioral health symptoms and HIV treatment engagement [16,17].

Consistent with the CETA emphasis on measurement-based care, CETA for people with HIV includes having patients complete standardized assessments on a routine basis for behavioral health symptoms, including a measure of depression (Patient Health Questionnaire–9) [18], generalized anxiety (Generalized Anxiety Disorder–7) [19], posttraumatic stress (Posttraumatic Stress Disorder Checklist-5) [20], and substance use (Alcohol, Smoking, and Substance Involvement Screening Test) [21]. We also piloted the use of a measure of HIV-related internalized stigma [22] at the first assessment to guide therapy planning and administered the measure as part of research surveys to explore the appropriateness for use as a standardized measure in a full-scale trial. Although not reassessed during CETA for people with HIV, demonstration of or increased risk of suboptimal HIV treatment engagement was assessed at baseline and defined as meeting at least one of the following criteria: (1) engaged in HIV care for the first time within the past 6 months, (2) having an HIV RNA viral load of >1000 copies/mL within the past 6 months, (3) having changed the antiretroviral regimen because of treatment failure in the past 6 months, or (4) having been a no-show to an HIV primary care appointment within the previous 12 months.

We maintained the original CETA structure of selecting and delivering therapeutic components designed to treat each clinical target and made two modifications to the components for people with HIV and HIV treatment engagement: (1) added a component to CETA for people with HIV that targets HIV treatment engagement based on an existing brief intervention called Life-Steps (described in the following section) and (2) added instructions to each CETA for people with HIV component as to how the skills taught in that component can be harnessed for addressing HIV-related stigma and barriers to HIV treatment engagement, if applicable (Textbox 1). For instance, to address stigma, patients can learn how to challenge negative unhelpful beliefs in the Thinking in a Different Way component and then apply these skills for modifying unhelpful self-stigmatizing beliefs about living with HIV. As an example of using skills for HIV treatment engagement, the Live Exposure component includes the example that patients may find themselves feeling anxious about and avoid going to their HIV treatment clinic and that the component can help them reduce this anxiety with practice. Finally, experts in behavioral health care for people with HIV and HIV treatment reviewed the CETA for people with HIV manual and added or modified examples for teaching skills to ensure that the examples were relevant to people with HIV.

##### Description of the Life-Steps Component

Life-Steps was selected as the modality for integrating HIV treatment engagement support into CETA for two primary reasons: (1) it has solid evidence demonstrating its effectiveness in improving HIV medication adherence and engagement, and (2) similar to CETA, it is based on principles of problem-solving therapy and cognitive behavioral therapy, facilitating its integration into the delivery of CETA [23]. Life-Steps was designed as a single counseling session for people with HIV to promote HIV treatment adherence. It has since been adapted for the HIV prevention context and is recognized by the Centers for Disease Control and Prevention as an evidence-based pre-exposure prophylaxis adherence intervention [24,25]. Life-Steps is based on principles of cognitive behavioral therapy, with an emphasis on problem-solving barriers to treatment engagement. Life-Steps assumes that there are several behaviors that individuals with HIV need to execute to be effectively engaged in treatment. These include having a medication-taking schedule, getting to appointments, having a plan for communication with health care providers, managing side effects, and having a plan for obtaining refills and storing medications. The Life-Steps component in CETA for people with HIV is consistent with previous iterations of this intervention. In CETA for people with HIV, it is delivered in the session following Encouraging Participation and Psychoeducation. The Life-Steps component covers the meaning of treatment engagement and its importance and helps patients explore the challenges they may face in being adherent to the various steps of HIV treatment. The clinician and patient develop a plan and a back-up plan for each step; however, more extensive work on complex barriers or those that require the patient to have more practice with cognitive behavioral skills taught in CETA for people with HIV (eg, problem-solving) is done as part of those relevant components. For instance, patients may first learn the problem-solving component of CETA for people with HIV and then apply these skills to specific barriers to attending HIV treatment visits.

#### Adaptation Step 2: Conduct Stakeholder Focus Groups to Inform Iteration of the Initial CETA for People With HIV Manual

##### Provider Focus Group

We conducted a focus group with 3 clinic social workers to obtain their feedback on the initial CETA for people with HIV manual; its conceptual approach to addressing behavioral health, HIV-related stigma, and HIV treatment engagement targets; and the appropriateness of the examples used to teach cognitive behavioral skills. The study coordinator emailed a total of 16 social workers at the clinic, and 3 (19%) agreed to participate in the focus group. All providers (3/3, 100%) self-identified as female, and 67% (2/3) identified as White. The mean age was 40 years (SD 13), with a total range of 25 years. The CETA for people with HIV study trainer and supervisor (MRS) led the provider focus group, which was conducted via the internet using videoconferencing software, recorded, and transcribed for analysis. Providers were given a copy of the introductory sections of the manual that included a description of the therapy, its structure, and the Encouraging Participation and Psychoeducation components to review ahead of time. The facilitator shared the contents of the manual being discussed during the focus group with the participants using videoconference technology.

##### Provider Focus Group Data Analysis

Transcripts of the focus group were generated and reviewed by coauthors DD, TRF, and MRS. The aim was to catalog the commentary made by participants on each question according to themes relevant to adapting CETA for people with HIV. Coauthors independently identified themes that came up in response to each focus group question and then met to discuss and come to a consensus on the themes. We present the findings according to the focus group questions (Textbox 2).

**Common Elements Treatment Approach (CETA) for people with HIV adaptation provider focus group guide.**

Purpose

- We conducted a focus group with clinic social workers to obtain their feedback on the initial CETA for people with HIV manual; its conceptual approach to addressing behavioral health, HIV-related stigma, and HIV treatment engagement targets; and the appropriateness of the examples used to teach cognitive behavioral skills.

Questions about the CETA for people with HIV approach to helping patients with behavioral health symptoms, stigma, and HIV treatment engagement

- How do you think CETA for people with HIV would help your patients?
- How well does the content related to HIV cover the kinds of concerns or problems clients have with their HIV treatment?

Questions relevant to select CETA for people with HIV components

- Encouraging participation
  This session of CETA for people with HIV introduces CETA for people with HIV to clients and addresses initial barriers to engaging in the therapy and program. What improvements can be made to engage people with HIV in CETA for people with HIV or address initial barriers common to people with HIV to engaging in session-based therapy?
- Life-steps, psychoeducation, and setting goals for HIV treatment adherence
  What psychoeducation or description of barriers to treatment engagement about living with HIV is missing from this section that we need to include?
  What steps (or aspects of treatment) that are critical in managing HIV care are not included that we need to include?
- Relaxation and live exposure
  What kinds of HIV treatment situations have you noticed clients feeling anxious about and possibly avoiding?
  What kind of anxiety-provoking situations that are unique to people with HIV do you commonly see in working with your clients?
- Getting active
  How might HIV contribute to people stopping activities that bring them pleasure, joy, a feeling of usefulness, or a feeling of being good at things?
- Thinking in a different way part I
  Please read the examples offered in the section for applying these skills to people with HIV. How might you improve upon or add to these examples based on experiences you have had working with people with HIV?
- Thinking in a different way part II
  Please read the examples offered in the section for applying these skills to people with HIV. How might you improve upon or add to these examples based on experiences you have had working with people with HIV?
- Substance use reduction
  What unique considerations to substance use and reducing substance use for people with HIV need to be added to this component?
  In what ways are the skills learned in this component useful for HIV treatment engagement?
- Safety
  What are examples of common suicidal thoughts related to HIV status?
  Are there any other safety concerns other than interpersonal violence, suicide, or homicide that should be included in this section? If so, what are they?
- Other
  What else would you like to comment on regarding CETA for people with HIV?

##### Patient Focus Groups

We conducted 2 focus groups with clinic patients to obtain their perspectives on how behavioral health symptoms and HIV-related stigma affect HIV treatment engagement and what they found helpful in addressing these issues. The study coordinator requested referrals for the patient focus groups from clinic social workers. A total of 9 patients were referred who met the eligibility criteria, and 7 (78%) agreed to participate when contacted by the study coordinator. To be eligible for participation in the patient focus groups, participants had to be current patients at the clinic (which also meant being HIV positive), aged ≥18 years, and willing to provide written informed consent. Although not an explicit inclusion criterion, the study team attempted to include as many patients as possible with experience of receiving treatment for behavioral health conditions. All but 1 participant (6/7, 86%) had experience with psychotherapy. On the basis of the demographics available in clinic records, most patients identified as female (5/7, 71%) and African American (6/7, 86%), and the mean age was 49 (SD 9; range 36-63) years. Patients’ experiences and perspectives were used to determine whether the initial CETA for people with HIV manual adequately reflected patient experiences and whether the therapeutic approach and manual content needed any modification.

We divided the patient participants into 2 groups—male (3/7, 43%) and female (4/7, 57%). As the HIV epidemic in the United States has different characteristics specific to sex, this division allowed patients to explore different experiences with their diagnosis among peers with similar experiences. The focus group primary facilitator was a clinical research manager at University of Alabama at Birmingham with experience conducting focus groups with clinic patients. In addition, a content expert was available during the focus groups to ask follow-up questions. Focus groups were conducted via the internet using videoconferencing software, recorded, and transcribed for analysis.

The focus group questions asked patients to comment on how 4 different behavioral health symptoms (anxiety, behavioral withdrawal, negative thoughts, and substance use) and HIV-related stigma affected HIV treatment engagement (Textbox 3) to observe themes that would influence modification of the Live Exposure, Getting Active, Thinking in a Different Way, and Substance Use Reduction components, including how to apply the skills in these components to address HIV-related stigma and HIV treatment engagement. We did not ask about all components in the interest of time and inquired about those components that we anticipated would be most commonly used.

**Common Elements Treatment Approach (CETA) for people with HIV adaptation patient focus group guide.**

Purpose

- We conducted 2 focus groups with clinic patients to obtain their perspectives on how behavioral health symptoms and HIV-related stigma affected HIV treatment engagement and what they found helpful in addressing these issues.

Questions about how behavioral health symptoms of anxiety, behavioral withdrawal, negative thoughts, and substance use as well as HIV-related stigma affect HIV treatment engagement

- Psychological symptoms that CETA for people with HIV helps people with are posttraumatic stress, depression, anxiety, and substance use problems.
  What are ways in which HIV status affects these symptoms based on your experience?
  How have these interfered with your ability to engage in or adhere to HIV treatment?
  What kinds of things have you tried to help with these symptoms?
- CETA for people with HIV helps people with HIV-related stigma.
  What kinds of experiences have you had with HIV-related stigma?
  In what ways has HIV-related stigma made it harder to engage in HIV treatment?
  What kinds of things have you tried to cope with HIV-related stigma?
- CETA for people with HIV helps people with HIV engage in HIV treatment.
  In what ways do you think it would be helpful to meet with a therapist weekly to help you better adhere to your HIV treatment plan?

Questions relevant to selected CETA for people with HIV components

- Live exposure
  What parts of your HIV care make you anxious?
  What parts of your HIV care do you find yourself avoiding because they make you feel anxious?
- Getting active
  What things have you stopped doing that may be related to your HIV status?
  Sometimes we also feel sad, down, or low. When this happens, we may also stop enjoying things we used to enjoy. Tell me how these feelings affect how well you take care of yourself, including your HIV.
- Thinking in a different way part II
  It is common for people with HIV to have negative thoughts about HIV treatment or their HIV status that can make it hard to engage in HIV treatment. What are some examples of these that you have had?
  What has helped you with these thoughts?
- Substance use reduction
  How does substance use affect HIV treatment? Substance use includes alcohol and other drugs that are nonprescribed or nonprescribed use of prescription drugs.
  What are some strategies you have used that have helped reduce the negative impact of substance use on HIV treatment engagement?
- Other
  What are other issues or barriers that patients face in improving their mental health or their HIV care engagement that we can add?
  What else would you like to share with me about what we have discussed regarding the study and the CETA for people with HIV therapy?

##### Patient Focus Group Data Analysis

The same coauthors (DD, TRF, and MRS) followed the analytical procedures used for the provider focus group. The aim was to catalog the commentary made by participants on each question according to themes relevant to patients’ experiences with behavioral health symptoms and stigma, how these affect their HIV treatment engagement, and how psychotherapy may be helpful in addressing behavioral health symptoms and stigma.

## Results

### Provider Focus Group Findings

#### CETA for People With HIV Structure

Providers were asked for their feedback on how components were structured in the manual, specifically by having the skills taught for behavioral health symptoms first followed by content on how to tailor the component and apply those skills specifically to people with HIV and help with their HIV treatment engagement. Providers liked this structure and commented that it may also help patients understand how their behavioral health symptoms are related to and interact with their experiences of living with HIV. A provider said the following:

I think that that is gonna be helpful in getting the patient to really think about where their anxiety or depression or where the posttraumatic stress disorder—where that’s actually coming from.

#### Common Problems With HIV Treatment Engagement and Life-Steps

Providers were asked whether the Life-Steps component adequately covered the common types of problems patients have that interfere with HIV treatment engagement. Providers confirmed that the problems identified in the component matched their experiences. They emphasized that patients have difficulty with consistent transportation to the clinic and may not be reachable for follow-ups. They noted that many patients do not have consistent phone service. Providers emphasized that most of their patients are low-income and living well below the federal poverty line and that many experienced housing instability and have lives that providers described as “chaotic.” They explained that it can be hard for patients to have the safety and stability in life to maintain consistency with treatment regimens and attend clinic appointments:

Sometimes, it’s really confusing ’cause you really believe this person really wants to take their medicine. They really wanna take every dose every day, but I think it goes back to that just livin’ in chaos and strugglin’ with the day to day.

Providers also noted that frustrations in navigating clinic procedures and interacting with other patients or staff can sometimes result in patients avoiding the clinic.

#### Behavioral Health Conditions Related to HIV Treatment Engagement

Providers confirmed that the manual adequately covered the common types of behavioral health concerns that contribute to HIV treatment engagement problems among their patients. Providers noted some unique considerations regarding the impact of behavioral health issues on HIV treatment engagement. First, they noted that receiving a diagnosis of HIV can create a sense of hopelessness and depression, particularly shortly after diagnosis, as patients may feel that their “life is over.” Providers also noted the different ways in which stigma specifically affects patients’ HIV treatment. For some patients, the fear of being recognized while waiting for their clinic visits prevented them from attending appointments. For other patients, the lack of a private place to store their antiretrovirals may also hamper their HIV treatment engagement. Thus, fear of accidental disclosure may keep patients from taking their medications if they are living in a situation in which they are not comfortable sharing their HIV status for fear of stigma or judgment:

Makin’ your appointments and takin’ your medicine every day just falls to the back. Well, and medicine, sometimes, is related to stigma, not havin’ a safe place where you can keep your medicine so nobody knows—people that you’re livin’ with don’t know that you’re HIV positive.

Providers expressed that substance use is common in their patient population and spoke about the different ways in which substance use deters HIV treatment engagement:

There are a lot of our patients who do struggle with substance use. For some, that can increase their anxiety, especially with...a lotta them have anxiety about comin’ to the clinic because of just them being seen at the clinic.

In addition, providers expressed that most of their patients would likely not pursue abstinence, making harm reduction an important approach, which might include strategies such as reducing the frequency and amount of use and using under safer circumstances (eg, clean needles for intravenous drug use). Finally, providers mentioned that their patients had experienced considerable trauma, and posttraumatic stress symptoms are frequently triggered by circumstances and interpersonal interactions at the clinic, which can result in avoidance of the clinic. They emphasized the importance of making trauma-informed care a clinic-wide effort.

### Patient Focus Group Findings

#### Barriers to HIV Treatment Engagement: Behavioral Health Conditions and HIV-Related Stigma

Patients were asked to comment on the role of behavioral health conditions and HIV-related stigma on HIV treatment engagement. Their comments indicated that these concerns have a negative impact on HIV treatment engagement. For instance, worries about taking medications contributed to not taking them as prescribed, such as potential side effects and loss of effectiveness and the discomfort of ingesting large pills. Patients expressed that depression symptoms such as behavioral withdrawal made it difficult to engage in care and take medications. They reported that substance use worsened urges to withdraw from others and avoid the clinic and negatively affected memory, decision-making, and general self-care (eg, sleep and hygiene), leading to inconsistent medication adherence and missed clinic appointments. A patient shared the following:

...well, specifically, depression, you have those days where you don’t want to do anything...for days where I didn’t wanna take a shower, get outta the bed. The last thing I was thinking about was taking medication.

Of note, patients commonly reported that behavioral health conditions were driven by being diagnosed as HIV positive, which was particularly the case shortly after diagnosis. Some patients reported that they felt depressed and hopeless after being diagnosed. Some patients also reported feeling that acquiring HIV was traumatic and they feared for their life after being diagnosed, leading to posttraumatic reactions:

For me, I went into a tailspin of depression. Before that, I don’t remember being depressed. You know what I’m saying? I know it was related to getting my diagnosis, and that’s when my life began to spiral out of control.

Patients described experiences with HIV-related stigma, including self-stigma and perceived and experienced stigma from other people, and reported that this was a barrier to engaging in HIV treatment. Common stigma experiences were fear of what others would think of them or how they would treat them if they knew of their HIV status, being rejected by friends and family, and having negative thoughts and feelings about themselves because of their HIV-positive status. Patients expressed stigma-related concerns regarding attending clinic appointments. Across the groups, anxiety about seeing acquaintances or people from their same social circles at their clinic was common. A participant said the following:

I didn’t want people to actually see me ’cause I was embarrassed. I still have that feelin’ sometimes, afraid of who you might see when you walk in ’cause people do gossip.

For some participants, religious affiliations of their own or among family proved problematic and potentially stigmatizing. A male participant noted the inability to disclose his HIV status because of his family’s religious affiliations:

Because my family was shunned away from me because of religious reasons and so forth. I couldn’t tell them. I couldn’t open up to them with anything about the HIV. I had to lie to them with it bein’ a cancer and stuff like that.

Although the male focus group had mixed, sometimes stigmatizing experiences related to religion, some female participants highlighted the positive aspects of religion and religious communities, including the solace found in their religious community, particularly after their initial HIV diagnosis. A female participant shared the following:

I started going to church more after that—after I found out I had it because anyway, the church members were prayin’ so hard for me, and I was so thankful for that.

Some participants also mentioned fear of and experiences with feeling judged or treated differently by clinic staff, including those providing care or administrative services. Patients also pointed out that HIV-related stigma is intersectional with social identity or demographic characteristics. For some patients, this meant the added stigma of being a part of multiple minority communities, such as being both HIV positive and Black in America. Patients also noted that some identities or demographic characteristics can interact with stigma. Men explored the ways in which gender and HIV diagnosis intersect. They shared how people in the Black community may view women living with HIV as victims whereas in turn assuming men became HIV positive because of having sex with other men in a way that can be perceived as careless:

I think it’s really different between men and women, especially with HIV care, ’cause I think that, for some people, they look at women, and they assume either that they were street workers, sex workers, or they were dealing with a man that had relations with another man. I think that gears towards the stigma as being a gay man’s disease.

Male participant

The interviewer reflected what the patient was saying, using the terms “victim” to describe women and “careless” to describe men to gain clarification from the participant. The interviewer’s word choices were confirmed by the participant:

Yeah, absolutely. The whole DL [“down low”] lifestyle and that kind of a big thing as far as—within the Black community, again, I think that that is really a perception.

Patients expressed that experiences dealing with stigma improved over time.

#### Therapy to Address Behavioral Health– and HIV-Related Stigma Barriers to HIV Treatment Engagement

Patients were asked to comment on how psychotherapy had helped them with behavioral health conditions and HIV-related stigma. All but 1 participant (6/7, 86%) had been in psychotherapy previously. Patients reported that psychotherapy had been helpful in addressing behavioral health conditions as well as anxiety about HIV-related stigma:

It’s giving me just an opportunity, really, to just address the traumas that I’ve experienced and just having someone to talk to. That has just been helpful in my day-to-day life, period, and just having a clear mind, not leaning on codependent relationships and substance abuse to probe.

A patient noted that having weekly therapy sessions helped them maintain stability in their HIV care as well:

I think, now that I have that care, and I have a routine, and I know that I’m working with an organization that cares about my overall wellbeing, it forces me to care about my own wellbeing. With goin’ to therapy, I adhere to my medication, and I believe I’m on the road to success to living a very normal life now.

Patients raised the concern that stigma related to experiencing a behavioral health condition is common, intersects with social identity, and negatively affects HIV treatment engagement. Patients acknowledged that it can be difficult to seek and engage in therapy as one does not want to be considered “crazy” or “weak” for needing this kind of service:

I think that, within our own community—maybe not so much that it has to be required, but maybe if there was a way to convey that therapy is helpful towards treatment, I think that that would be a great step in the right direction ’cause I think that that is a huge stigma in [the Black] community is that we believe that therapy is for the weak or for the crazy.

Male participant

### Adaptation Step 3: Revise the Initial CETA for People With HIV Manual Based on Provider and Patient Focus Groups

DD, MRS, and CP met to review the focus group findings and discuss adaptations to make to the initial CETA for people with HIV manual. We determined that the observed themes and experiences illustrated in the focus groups were adequately captured in the initial manual and that no substantive changes were needed.

### Adaptation Step 4: Train Study Counselors in CETA for People With HIV and Pilot the Manual With Training Cases

#### Training Cases

The study team piloted the CETA for people with HIV manual with the first 3 participants who were randomized to CETA for people with HIV therapy as part of a pilot clinical trial currently underway. These participants provided case-based training for counselors to hone their ability to deliver the CETA for people with HIV intervention. Participants were referred for the study by clinic providers (Medical Doctors and Nurse Practitioners) and social workers and met with the study coordinator to be screened for the study. The inclusion criteria were being aged ≥18 years; receiving HIV care at the study clinic; having elevated symptoms of depression, anxiety, posttraumatic stress, or substance use problems; and being at risk of suboptimal HIV care engagement (having at least one of the following: engaged in HIV care for the first time within the previous 6 months, had an HIV RNA viral load of >1000 copies/mL within the previous 6 months, changed antiretroviral regimen because of treatment failure within the previous 6 months, or been no-shows to an HIV primary care appointment within the previous year). Prisoners and those unable to provide informed consent were excluded.

#### CETA for People With HIV Workshop Training

An expert CETA trainer (MRS) led a training via the internet with the study counselors in July 2020. The Life-Steps component was led by an expert in the delivery of this intervention (CP). The training was planned to be in person; however, because of the COVID-19 pandemic, it was held via the internet over 4 half days. Trainees received a copy of the CETA for people with HIV manual describing the rationale for and how to perform the steps of each component. The training used didactics, videos demonstrating CETA skills, an exercise to practice selection of CETA for people with HIV treatment flows (ie, plans) and clinical targets, and role-play practice with trainer feedback in delivering CETA for people with HIV components.

#### Postworkshop Consultation

A total of 3 study counselors (one was the primary study counselor who saw most cases) completed a training workshop followed by twice-monthly consultation calls, and each was assigned a training case. One counselor left the clinic after the first session with her training case, which was reassigned to the primary study counselor.

Following the workshop, and for the first 2 months of the study, the CETA for people with HIV trainer led twice-monthly consultations via videoconference with study clinicians. Consultation sessions were scheduled for an hour and provided opportunities to rehearse, review, and expand on CETA for people with HIV delivery skills. Similar to the workshop, the trainer used multiple instructional methods such as reviewing the manual, practicing skills, having counselors practice CETA for people with HIV skills using their own life experiences, and discussing clinical examples. Moreover, because of the COVID-19 pandemic, all study patients had the option to have telehealth visits. Therefore, consultation sessions included instruction on how to use telehealth and videoconferencing technology for CETA for people with HIV as this was a new mode of treatment delivery for the counselors. Instruction was provided on how to prompt patients to follow along in sessions with their patient materials, how to use screen-sharing features to review handouts and worksheets, and how to manage potential technical difficulties.

#### CETA for People With HIV Supervision

Once counselors started seeing training cases in February 2021, the trainer held twice-monthly group supervision that ranged from 1 to 1.5 hours with the 2 counselors to promote fidelity to the CETA for people with HIV treatment model. This became weekly when consultation sessions ended in April 2021. Supervision was supported by an electronic data capture system that counselors used to document their CETA for people with HIV flow, clinical targets, symptom assessments, and session activities, which could be reviewed by the supervisor before or during supervision. Supervision also included an in-depth discussion of the CETA for people with HIV treatment flow, the implementation and dosage of CETA for people with HIV components to reduce symptoms for clinical targets, review of patient progress in acquiring and practicing CETA for people with HIV skills, and review of symptom assessment scores for clinical targets.

#### Posttraining Manual Modifications

The only change made to the manual after counselors completed training cases was to design a step sheet for the Life-Steps component that details what the counselor should do in the Life-Steps component. As there were no substantial changes made to the CETA for people with HIV manual or the nature and structure of the consultation and supervision provided to counselors, the 3 participants randomized in step 4 were included in the larger pilot, described in step 5.

### Adaptation Step 5: Pilot the CETA for People With HIV Intervention

#### Overview

A pilot clinical trial with 60 patients at the clinic is underway that aims to assess the acceptability, feasibility, and fidelity of CETA for people with HIV as well as a signal of clinical impact, such as behavioral health and HIV treatment engagement metrics. Participants are randomized 1:1 to CETA for people with HIV or treatment as usual. Counselors trained in CETA for people with HIV for the training cases remained counselors throughout the pilot trial.

#### Use of Telehealth to Implement CETA for People With HIV

The beginning of the COVID-19 pandemic coincided with the start of the CETA for people with HIV training and pilot trial. To accommodate telehealth visits, the study team purchased and distributed tablets enabled with videoconferencing and built-in wireless capabilities and provided access to a web-based survey platform to complete symptom assessments in a deidentified fashion. All other capabilities on the tablet were disabled. Counselors and study staff offered to coach patients on how to use the technology. Patients could also have telephone appointments if they were unable to use videoconferencing. Later, when patients were able to return to in-person visits at the clinic, they had the option of attending sessions in person, by telephone, or by tablet-facilitated videoconference. Patients were provided with a hard-copy workbook with handouts that they would need for CETA for people with HIV to take home. The study team observed early challenges with the technology, such as lost or stolen tablets, lack of local services to support the built-in wireless capability, and lack of privacy to hold telehealth sessions. In contrast, the team observed that tablets allowed some participants to engage in treatment who would have had substantial difficulty attending a weekly in-person appointment. This pilot trial will shed light on the feasibility of using this technology with patients.

### Case Example

#### Overview

Patients receiving care at the HIV clinic face many psychosocial stressors that serve as barriers to engaging in and completing treatment, which requires support from clinic case managers and other providers. It also required flexibility on the part of CETA for people with HIV counselors to be able to reschedule appointments, including early-morning or late-afternoon appointments, and accommodate the constantly changing needs of patients. CETA for people with HIV therapy flows can be flexibly modified to teach the skills needed for patients’ most emergent HIV treatment barriers. The case example in the following sections illustrates a completed course of CETA for people with HIV facilitated by the counselor’s ability to help with transportation, be flexible in scheduling, and responsively modify the order of planned therapeutic components based on the patient’s progress through therapy.

#### Demographic and Clinical Information

Patient A enrolled in the pilot clinical trial currently being conducted. He is an African American, middle-aged gay male individual. He is a high school graduate who was unemployed at the time he began the CETA for people with HIV intervention. During his time in the study, he faced several psychosocial stressors. Owing to unemployment and limited financial resources, patient A was often uncertain about being able to pay rent, lived without utilities for a period, and experienced food insecurity resulting in days without eating. Once assigned to a case manager at the clinic, many of these stressors were resolved. He obtained assistance with food benefits and support with rent and utilities. Later, patient A obtained employment, which substantially affected his financial situation in a positive way.

Patient A was diagnosed with HIV within the year before starting the CETA for people with HIV intervention and qualified for the study because of elevated mental health symptoms (posttraumatic stress, anxiety, and depression), moderate alcohol and marijuana use, and missed HIV care appointments. Patient A’s social support network consisted of a close male friend with whom he was sexually intimate and the patient’s siblings. His supports were not aware of his HIV status because of his fear of rejection. His reasoning for not sharing his HIV status with his friend and sexual partner was that his viral load was undetectable and, therefore, would not be transmitting HIV.

#### CETA for People With HIV Therapy Flow

Patient A and his clinician agreed to target symptoms of anxiety, posttraumatic stress, and HIV-related stigma in addition to completing the Life-Steps component to address HIV treatment engagement concerns and missed appointments. This included teaching skills from the Relaxation, Thinking in a Different Way Parts I and II, and Live Exposure components.

#### Therapeutic Engagement

Attendance at therapy and HIV medical appointments was not initially problematic because of the use of problem-solving skills taught in Life-Steps. Patient A lacked access to transportation and, at the beginning of therapy, was awaiting assignment of a case manager who could assist with scheduling transportation assistance for appointments at the clinic. The clinician and patient A were able to create a plan to connect patient A with the study research coordinator for assistance with scheduling transportation to appointments. Although the patient initially used telehealth appointments for the CETA for people with HIV intervention, which helped with the lack of transportation, he switched to in-person appointments later in the study because of preference, and scheduled transportation was very helpful for coming to in-person sessions.

Patient A was highly engaged in the therapy. He came to sessions prepared with his therapy manual, actively participated in learning new skills in the session, and practiced those newly learned skills in between sessions for skill practice homework. Patient A regularly completed homework worksheets and brought them to sessions for review with his clinician. Toward the end of treatment, patient A found employment and often needed to reschedule appointments; a few times, he missed appointments entirely. He continued to prioritize therapy and work toward completion as much as possible. Although the completion of skill practice worksheets decreased, patient A continued to practice skills. He reported that he usually mentally practiced skills between sessions, often during free time such as riding the bus to or from work.

Patient A completed CETA for people with HIV and participated in a total of 13 sessions over a period of 4 months. There were 4 sessions that needed to be rescheduled because of patient scheduling conflicts and 3 missed appointments. Most cancellations and missed appointments occurred later in the treatment, when patient A had obtained employment and his work hours conflicted with scheduled appointments.

#### Use of Therapeutic Components

As part of Thinking in a Different Way Part I, the counselor taught patient A the skill of identifying negative unhelpful thoughts in response to everyday situations and generating more helpful thoughts in response, which in turn decreases negative emotion. Patient A reported that he often found himself having such negative or unhelpful thoughts. Through therapy, he learned to slow down his thinking, identify how a thought was unhelpful, and formulate more helpful alternate thoughts. Without much instruction, patient A was able to naturally progress from this skill to those taught in Thinking in a Different Way Part II, which focuses on challenging negative unhelpful beliefs about the self, other people, or the world.

A negative belief that the patient had was blame for the death of a relative, which contributed to posttraumatic stress symptoms. However, he required minimal sessions dedicated to Thinking in a Different Way Part II to address this belief because of an unexpected naturalistic opportunity to challenge this belief through Live Exposure that occurred in his life. Specifically, the patient found himself in a situation that was similar to the one he was in when his relative died, and he was able to see that he was not to blame for what happened. In addition, although highly triggering, using Relaxation skills and Thinking in a Different Way Part I skills to reduce physiological arousal and coach himself through the stressful situation, patient A was able to successfully approach the experience, which also helped reduce negative unhelpful trauma-related beliefs about himself. Patient A so successfully approached this situation and generalized learning from the experience that he did not need to then have additional sessions of Live Exposure.

The patient’s experience of stigma was about discomfort being seen at the clinic by someone he knew and concern about being judged by them. During the study, the clinic moved from a previous location with signage reading “Infectious Disease” and “Psychiatry.” The new clinic location had discreet signage that did not indicate why a patient might be seen there, which helped the patient feel more comfortable coming to appointments.

#### Therapeutic Outcomes

Overall, there were several improvements observed in the therapy. Patient A did not miss any HIV care appointments, and his viral load remained undetectable. Patient A then reported that stigma about coming to the clinic because of signage and location had ceased. The patient’s self-reported symptoms on standardized measures for posttraumatic stress and anxiety dropped to subclinical levels.

## Discussion

### Principal Findings

CETA was readily adapted as a brief cognitive behavioral therapy to support engagement in HIV treatment for people with HIV in a comprehensive HIV treatment clinic. Focus groups with clinic social workers and patients who had experience with behavioral health conditions and psychotherapy indicated that the adapted therapy made conceptual sense and addressed common behavioral health concerns as well as practical and cognitive behavioral barriers to HIV treatment engagement.

A methodological limitation of the adaptation process was that the sample sizes of the focus groups were small and not representative of the full spectrum of social workers or patients at the clinic. Some restrictions were intentional, for example, the patient focus group purposively recruited patients with a history of treated behavioral health conditions and experience with psychotherapy to benefit from their lived experiences of how behavioral health conditions affect HIV treatment and how psychotherapy could help. However, the experiences of these patients may not correspond to the experiences of the patients who end up in the pilot trial, who may never have engaged in a session-based therapy previously and may be earlier in their postdiagnosis period. Despite the small sample sizes, we are encouraged by the fact that we identified elements similar to those identified by other researchers for successfully engaging people with HIV in a transdiagnostic psychotherapy [26].

Completing the adaptation steps as we described with stakeholder input does not guarantee an adapted therapy that will be effective across all clinic patients as the perspectives of the experts, counselors, and patients engaged in the adaptation may be biased in ways that do not reflect the experience of the larger population. Pilot-testing and then effectiveness testing of the adapted therapy are important next steps in the adaptation process. Our team is currently piloting the adapted therapy and will collect quantitative and qualitative data from a more representative sample of clinic patients to make further refinements before completing a definitive trial of the therapy.

### Considerations for CETA for People With HIV and HIV Treatment Engagement

Key considerations for CETA for people with HIV obtained from the focus groups were related to stigma, socioeconomic stress and instability experienced by the population (also illustrated in the case study), and the use of substances by some patients, which can thwart the stability needed to engage in care. Stigma related to HIV, behavioral health conditions, and receiving care for each of these is well documented in the research literature [27,28] and was reflected in participant responses to focus group questions. For example, some participants mentioned fear of seeing someone they knew at the clinic and the potential for accidental disclosure or gossip in their shared community. Some mentioned feeling judged by a few of the clinic staff members or providers. These experiences could unfortunately lead to missed therapy appointments and limit the opportunity to help patients navigate their experiences of stigma. These experiences also point to the importance of supporting staff in their skills for working with people with HIV and behavioral health conditions and clinical settings being mindful of aspects of their physical environment that could contribute to stigma.

Experiences of HIV- and behavioral health–related stigma are known to intersect with other social and cultural identities such as gender, race, ethnicity, and sexual orientation, which can compound challenges in accessing care [29]. Participants in all 3 focus groups explained how being a Black gay man in America comes with layers of stigma and, further, how these various stigmas intersect and create barriers for mental health and HIV care for Black men in particular.

Among female participants, many described the support they found in their churches or religious communities when they received their HIV diagnosis. However, support from religious communities may not translate to all patients of the clinic, especially for those who identify as lesbian, gay, bisexual, transgender, and queer (LGBTQ+). Taggart et al [30] note the importance of understanding the messages of LGBTQ+ behaviors communicated in religious or spiritual settings as it is possible that religion or spirituality may moderate a path between a person’s internalized HIV stigma and depression. Although this could be harmful for some patients, the authors noted that, for some people with HIV, their experiences with religion and spirituality appear to be protective (eg, lessening the association between internalized HIV stigma and depression). Female participants in this study mentioned their religion or spirituality as a means of support after their HIV diagnosis. This theme did not emerge in the male group; rather, they discussed being disowned by family members because of religious reasons as well as a need to hide their sexual and HIV identities from family members who attended church. Previous research among young Black gay men has highlighted experiences of homophobia and HIV-related stigma from churches and families within the Black community and friends in the Black gay community who typically provide support to these men as they face racism [31]. Disparity in psychological well-being among people with HIV who identify as LGBTQ+ has been well documented [32], making interventions for the LGBTQ+ community of critical importance.

Understanding the nuances of intersectional stigma is crucial to providing thoughtful and holistic care for marginalized communities, especially communities with long histories of barriers to care in the United States. The ongoing pilot trial of CETA for people with HIV aims to shed light on how to support patients in addressing stigma related to HIV and behavioral health care; however, we recognize that therapy cannot address systemic issues in this country or correct for the range of injustices that people with HIV and those with behavioral health conditions face each day, particularly if they are patients of color. Therapy does not change the larger social norms, but it can acknowledge these injustices, support and validate patients, and offer tools for problem-solving and coping to help patients with their experiences of stigma and injustice.

Challenges related to income, housing, and psychosocial concerns are commonly experienced by patients at the clinic. Although CETA for people with HIV can help problem-solve and address these barriers to HIV treatment, it requires that patients be able to engage routinely in session-based therapy—either via telehealth or in person. The barriers to HIV treatment may also thwart patients’ efforts to engage in CETA for people with HIV. A consideration is that patients enrolled in session-based therapy such as CETA for people with HIV may require simultaneous case management to help them handle ongoing needs for housing and employment and access social services. For the pilot study of CETA for people with HIV, patients continue to work with their HIV clinic case manager. In addition, patients in the focus group mentioned the value of psychotherapy in providing a structure and routine that supported HIV treatment engagement. It may be important for case management to provide this structure in an ongoing manner given that CETA for people with HIV is a time-limited therapy.

Regarding substance use concerns, social workers in the provider focus group mentioned that substantially reducing substance use or abstinence may not be reasonable goals for many of their patients and that a harm reduction approach [33] may be most appropriate. For example, a harm reduction approach could help patients reduce the negative impact of substance use on taking medications or attending clinic appointments by helping them identify ways to change when they use even if patients do not reduce how much they use overall. The Substance Use Reduction component combined with the Life-Steps component is expected to support patients in this way; however, the pilot study currently underway may shed light on whether additional changes are needed or resources need to be added to CETA for people with HIV to better engage patients in a harm reduction model.

A consideration that emerged through the focus groups was the timing of the occurrence of an HIV diagnosis. Research suggests that patients shortly after diagnosis are particularly vulnerable to increased rates of depression [34], which could lead to care disengagement. Being diagnosed earlier in the epidemic meant having limited treatment options; however, there are now >30 individual medications available to treat HIV, many of which are available in single-pill formulations. It may be that, once engaged in treatment, recently diagnosed people with HIV experience more hope about living a full life with HIV. Qualitative data among older women living with HIV suggest that some individuals with HIV “adjust” to their diagnosis and develop self-efficacy regarding illness management over time [35], facilitating effective care engagement. Patients in our focus groups mentioned resonant experiences and indicated that the immediate negative impacts of being diagnosed improved over time. However, how newly diagnosed patients view their diagnosis relative to advances in HIV treatment and improvements in life expectancy and their impact on care engagement remains unknown. Therefore, it is critical to offer psychosocial support to patients at the time of diagnosis and throughout illness management.

### Considerations for Training and Delivery of CETA for People With HIV During the COVID-19 Pandemic

The research team was able to rapidly shift from in-person to internet-based formats for the training and delivery of CETA for people with HIV. The use of a videoconferencing platform to deliver instruction and internet-based tools such as breakout rooms and whiteboards to support skill practice allowed the trainer to mirror an in-person training experience. Internet-based phone or videoconference-based supervision and consultation were an accepted practice before the COVID-19 pandemic, and the trainer was able to routinely engage with the counselors through this medium.

Owing to COVID-19, the clinic shifted to a temporary telehealth-only model for counseling services. The study team also adapted, purchasing tablets with built-in internet for patient use and web-based data collection systems for completing symptom measures. Despite the effort to make internet-based CETA for people with HIV visits available, we observed some barriers to their use for psychotherapy, which may reflect the psychosocial circumstances of the patient population. For instance, some patients do not have private spaces in their homes from which to attend internet- or phone-based appointments, which may be more likely for our patient population, in which housing instability is common. Some patients were also not able to obtain a signal for the tablet’s built-in wireless internet [36]. Once in-person visits were allowed again, patients were offered in-person, phone, or internet-based visits. The findings from our pilot study will shed light on whether patients were able to consistently engage through these methods and how best to engage our patient population in consistent, accessible in-person or internet-based visits.

### Conclusions

Our team successfully used principles of the ADAPT-ITT framework to adapt CETA for people with HIV. The resulting brief, manualized therapy is designed to help patients build skills that promote HIV treatment engagement as well as reduce symptoms of common behavioral health conditions that themselves are known to thwart HIV treatment engagement. The research team is completing pilot research that will help determine whether the adaptations lead to a feasible and acceptable model to meet the needs of patients receiving care in US-based HIV clinics.

## Abbreviations

ADAPT-ITT: Assessment, Decision, Administration, Production, Topical Experts, Integration, Training, Testing
CETA: Common Elements Treatment Approach
LGBTQ+: lesbian, gay, bisexual, transgender, and queer
LMIC: low- and middle-income country

## Data Availability

Data sharing is not applicable to this study as no quantitative data sets were generated or analyzed.
